# Pediatrics

**Published:** 1906-07

**Authors:** Maud J. Frye

**Affiliations:** Buffalo, N. Y.


### PROGRESS IN MEDICAL SCIENCE.

### Pediatrics

Conducted by MAUD J. FRYE, M. D., Buffalo, N. Y.

### THE BUDDE PROCESS FOR PRESERVING MILK

HEWLETT (*Lancet,* January 27, 1906) describes the process as follows: The milk is obtained in as cleanly condition as possible and if it has to be kept anytime before treatment it is effectively chilled. A proper proportion of peroxide of hydrogen is added to the milk and the mixture is heated to from 51° to 52°c. for at least three hours. A temperature below 48°c. is not efficient and one above 55° tends to induce changes in the milk. With the aid of the heating the hydrogen peroxide is completely decomposed into water and oxygen by an enzyme (catalase) present in the milk and the nascent oxygen acts as an efficient germicide. At the end of the process the whole of the peroxide should have been decomposed, so that no antiseptic remains, a small but inappreciable amount of water has been added to the milk, and the majority of microorganisms have been destroyed. The amount of hydrogen peroxide required is about 15cc. of a 3% solution for a litre of milk. The milk is treated in bulk and is immediately bottled. It is practically unaltered in appearance and flavor; the cream rises as usual; the acidity is not increased, and the milk will keep sweet for at least eight or ten days in hot weather. All the spore-bearing organisms tested, pathogenic and non-pathogenic—the b. tuberculosis, the b. diphtheriæ, the b. acidi lactici, the b. typhosus, the b. coli, the b. dysenteriæ, a paratyphoid bacillus, the micrococcus pyogenes aurens and the cholera spirillum—are destroyed by the process. Sporulating forms—the b. arthracis, the b. subtilis, the b. mycoides, and the penicillium glaucum—are not destroyed, the inference being that vegetative forms are destroyed but the spores are not destroyed. Although the heating per se may be efficient in destroying certain non-sporulating organisms, it is not so in all cases. "Buddeising" is far more effective.—*Abstract in Am. Journal of Obstetrics.*

---

### COLLARGOL IN PUERPERAL SEPSIS

Edward Speidel, Professor of Obstetrics and Diseases of Women, Hospital College of Medicine, reported before the Louisville Medical and Surgical Society, Nov. 21, 1904, an extreme case of puerperal sepsis in which he had excellent results with collargol. After two weeks' treatment with the remedy the patient had a normal temperature and became convalescent. He does not like the use of streptolytic serum, which he employed in several cases, using seven bulbs in one without benefit.—*American Practitioner and News.*

